# Development and assessment of a novel gold immunochromatographic assay for the diagnosis of schistosomiasis japonica

**DOI:** 10.3389/fimmu.2023.1165480

**Published:** 2023-04-03

**Authors:** Yi Mu, Donald P. McManus, Catherine A. Gordon, Hong You, Allen G. Ross, Remigio M. Olveda, Pengfei Cai

**Affiliations:** ^1^ Molecular Parasitology Laboratory, QIMR Berghofer Medical Research Institute, Brisbane, QLD, Australia; ^2^ Rural Health and Medical Research Institute, Charles Sturt University, Orange, NSW, Australia; ^3^ Department of Immunology, Research Institute for Tropical Medicine, Manila, Philippines

**Keywords:** schistosomiasis, *Schistosoma japonicum*, GICA strip, lateral flow immunochromatographic test, ELISA, rapid diagnosis, point-of-care (POC), surveillance

## Abstract

**Background:**

The neglected zoonosis, schistosomiasis japonica, remains a major public health problem in the Philippines. The current study aims to develop a novel gold immunochromatographic assay (GICA) and evaluate its performance in the detection of *Schistosoma japonicum* infection.

**Methods:**

A GICA strip incorporating a *S. japonicum* saposin protein, SjSAP4 was developed. For each GICA strip test, diluted serum sample (50 µl) was loaded and strips were scanned after 10 min to convert the results into images. ImageJ was used to calculate an R value, which was defined as the signal intensity of the test line divided by the signal intensity of the control line within the cassette. After determination of optimal serum dilution and diluent, the GICA assay was evaluated with sera collected from non-endemic controls (n = 20) and individuals living in schistosomiasis-endemic areas of the Philippines (n = 60), including 40 Kato Katz (KK)-positive participants and 20 subjects confirmed as KK-negative and faecal droplet digital PCR assay (F_ddPCR)-negative at a dilution of 1:20. An ELISA assay evaluating IgG levels against SjSAP4 was also performed on the same panel of sera.

**Results:**

Phosphate-buffered saline (PBS) and 0.9% NaCl were determined as optimal dilution buffer for the GICA assay. The strips tested with serial dilutions of a pooled serum sample from KK-positive individuals (n = 3) suggested that a relatively wide range of dilutions (from 1:10 to 1:320) can be applied for the test. Using the non-endemic donors as controls, the GICA strip showed a sensitivity of 95.0% and absolute specificity; while using the KK-negative and F_ddPCR-negative subjects as controls, the immunochromatographic assay had a sensitivity of 85.0% and a specificity of 80.0%. The SjSAP4-incorperated GICA displayed a high concordance with the SjSAP4-ELISA assay.

**Conclusions:**

The developed GICA assay exhibited a similar diagnostic performance with that of the SjSAP4-ELISA assay, yet the former can be performed by local personnel with minimal training with no requirement for specialised equipment. The GICA assay established here represents a rapid, easy-to-use, accurate and field-friendly diagnostic tool for the on-site surveillance/screening of *S. japonicum* infection.

## 1 Introduction

In the recently released new WHO roadmap for neglected tropical diseases (NTDs) 2021-2030, it has been emphasized that effective diagnostics are key components of NTD programmes, from confirmation of disease to mapping, screening, surveillance, monitoring and evaluation, representing a prerequisite for reaching the 2030 disease targets (1). Schistosomiasis, one of the major NTDs, affects more than 250 million people worldwide (2). Six species of schistosomes (trematode blood flukes) infect human beings, of which *Schistosoma mansoni*, *S. haematobium*, and *S. japonicum* are the major species. The control of the disease relies heavily on mass drug administration (MDA) employing praziquantel (PDZ) (3). Recently, both the prevalence and infection intensity of the disease have declined in many endemic areas due to the implementation of integrated control programs, including MDA, making conventional parasitological diagnostics (e.g., the microscopy-based Kato Katz (KK) and the miracidium hatching technique) less efficient in the detection of schistosomiasis. These situations thus necessitate the development and implementation of more cost-effective and accurate diagnostics for rapid mapping and monitoring schistosomiasis in the endemic areas.

In addition to conventional parasitological diagnostics, there are a number of other diagnostic methods available for the detection of schistosome infections (4–8). Improved coprological tests, such as the saline gradient method (9), formalin-ethyl acetate sedimentation-digestion (FEA-SD) (10) and Helmintex method (i.e., isolates eggs from fecal samples with the use of paramagnetic particles in a magnetic field) (11) showed an increased diagnostic sensitivity compared with the traditional techniques; yet are usually labour-intensive and have a lengthy processing time. In addition, polymerase chain reaction (PCR) technology-based molecular diagnostics, including real time quantitative (q)PCR- (12–15), droplet digital (dd) PCR-based assays (16–18), loop-mediated isothermal amplification (LAMP) (19–21), and recombinase polymerase amplification (RPA) (22–25) are alternative tools for schistosomiasis diagnosis due to their outstanding accuracy; however, these tests require experienced human resources and are expensive (e.g. the relatively high cost of DNA extraction, qPCR reagents and/or equipment), limiting their application in remote areas with limited resources.

Point-of-care (POC) diagnostics are at the forefront of government initiatives, non-government organisations, medical diagnostics companies as well as fundamental research (26). For NTDs, accurate POC testing facilitates rapid results *in situ*, enabling targeted intervention for drug treatment and providing insights into dynamic transmission of pathogens and the burden of diseases. The application of POC tests can increase acceptance of treatment and minimise the risk of potential development of drug resistance. Recently, the WHO called for expert consultation on diagnostics with a particular focus on POC testing for schistosomiasis and soil-transmitted helminths (27). As a powerful POC tool, immumochromatographic assay (ICA) has been widely developed with a particular application in epidemiological surveys in the field. Recently, a number of colloidal gold immunochromatographic assay (GICA) strips have been established and evaluated for the rapid diagnosis of parasitic diseases, such as schistosomiasis (28–31), toxoplasmosis (32), fascioliasis (33) and opisthorchiasis (34), as an alternative screening tool.

Current antigen detection (AgD)-based POC tests schistosomiasis are based on the probing proteoglycan components present in the gut vomit of juvenile and adult worms known as circulating anodic antigens (CAAs) or circulating cathodic antigens (CCAs) using lateral flow assays (2). The POC-CCA is a commercially available cassette assay that has been widely validated in the detection of active *S. mansoni* infection in high and moderate endemic areas. However, the assay showed lower potential in the diagnosis of other *Schistosoma* species (35–37). In addition, the assay suffers some pitfalls, such as cross-reactivity, underperformed specificity, and a ‘Trace’ reading problem (36, 38). To date, the antibody detection (AbD)-based GICA assays developed for schistosomiasis japonica diagnosis typically use crude worm or egg antigen, which can result in cross-reactions with other helminths during field clinical sample testing. Previously we, and others, identified a schistosome saposin protein, SjSAP4, which shows an unprecedented level of accuracy for diagnosing infected cohort subjects from *S. japonicum*-endemic areas in both China and the Philippines (39–41). In this study, we aimed to develop and assess a novel SjSAP4-incorporated GICA strip for the diagnosis of schistosomiasis japonica.

## 2 Methods

### 2.1 Ethics

The human research ethical approval for the study was obtained from the Human Research Ethics Committee, QIMR Berghofer Medical Research Institute (QIMRB), Brisbane, Australia (Project Approval: P524) and the Institutional Review Board (IRB) of the Research Institute for Tropical Medicine (RITM), Manila, Philippines (IRB Number 2015-12). Written consent was obtained from all participants (for children aged 15 years or under written consent was obtained from their legal guardians).

### 2.2 Study cohort, sample collection, processing, and storage

The study recruited human subjects from 18 barangays moderately endemic (27% prevalence) for schistosomiasis japonica in the municipalities of Laoang and Palapag, Northern Samar, Philippines, in 2015 (3, 18, 39, 42). Fecal and serum samples were collected from the participating subjects. For each participant, two fecal samples (10–15 g each) were sought on different days within a week for the KK analysis. After fixing in 80% ethanol, the remainder of the first fecal sample (~10 g) was stored at 4°C. A blood sample (10 ml) was collected from each individual using a 10 ml serum silica vacutainer. The blood sample was set to clot at ambient temperature for 30 min. After centrifugation at 1500 × *g* for 10 min, the serum samples were aliquoted. All clinical samples were kept at 4°C and transported on wet ice to the RITM, where the samples were stored at -20°C. All samples were subsequently shipped to QIMRB, Brisbane, Australia on dry ice. In this study, a subset of serum samples (n = 60) collected from the endemic areas was tested with the GICA strips. Serum samples collected from healthy human subjects (n = 20) living in a non-endemic area (Qiqihar, Heilongjiang Province, China) for schistosomiasis served as controls.

### 2.3 Parasitological detection (Kato-Katz)

Kato-Katz analysis on fecal samples was performed by experienced technicians at RITM. For each fecal sample, three KK slides were examined. Infection burden was presented as the number of eggs per gram of feces (EPG). To increase the accuracy of the KK test, 10% of slides were randomly selected for re-examination by an experienced microscopist.

### 2.4 Cloning, expression and purification of recombinant SjSAP4

A gene fragment of SjSAP4 (GenBank No: ON241030, nt 1-534) with an additional pET28a vector-derived sequence -CCATGGGCAGCAGCCATCATCATCATCATCAC- at N-terminal was synthesized. The DNA fragment was amplified by PCR from the synthetic gene with forward primer: AACCATGGGCAGCAGCCATCAT and reverse primer: AACTCGAG-TAATGGACACA-ACTGTATTG. The PCR product was purified and further digested with restriction enzymes *Nco*I and *Xho*I, and the DNA fragment was then cloned into the pET-28a vector. Recombinant plasmid was confirmed by DNA sequencing and transformed into Rosetta (DE3) competent cells. Expression of the recombinant SjSAP4 (rSjSAP4) protein was induced by 0.2 mM IPTG. The rSjSAP4 protein was purified under native conditions using Ni-IDA Sepharose Cl-6B (Novagen, San Diego, CA, USA) according to the manufacturer’s instructions. The purified rSjSAP4 protein was analyzed by 12% (w/v) SDS-PAGE and Western blot analysis (**Supplementary Figure S1**).

### 2.5 Preparation of the GICA strips

**Figure 1:**
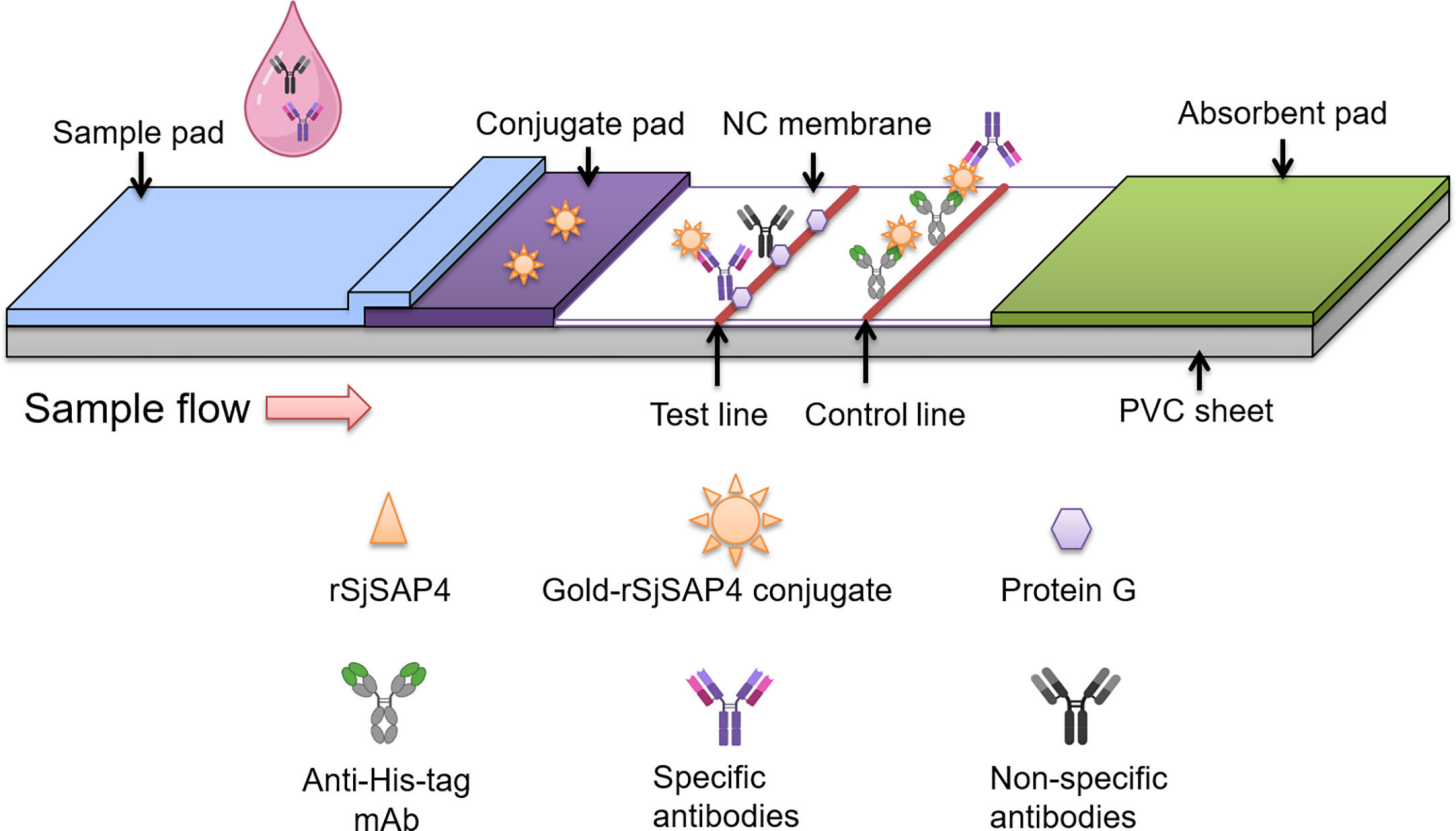
Schematic illustration of the rSjSAP4-incorporated GICA strip. The gold-rSjSAP4 conjugate is added to the conjugate pad. The recombinant protein G and a monoclonal mouse anti-His tag antibody is immobilized on the test (T) and control (C) line, respectively, on the NC membrane. Once serum samples containing specific anti-SjSAP4 antibodies or non-specific antibodies loaded onto the sample well, the conjugated anti-SjSAP4 antibody complexes and non-specific antibodies are captured by the protein G immobilized on the ‘T’ line. The gold-rSjSAP4 conjugate and conjugated anti-SjSAP4 antibody complexes are captured by the anti-His tag antibody on the ‘C’ line. A positive result is indicated by the appearance of pinkish red bands on both the ‘T’ and ‘C’ lines. A negative result is indicated by the appearance of only a single pinkish red band on the ‘C’ line. A test is determined as invalid if no bands appear on both ‘T’ and ‘C’ lines or if only one band appears on the ‘T’ line, and thus needs to be re-tested.

The GICA strips were developed by ZoonBio Biotechnology (Nanjing, China). The design of the GICA strip was illustrated in **Figure 1**. Briefly, rSjSAP4 protein was initially coated with colloidal gold particle (70 nm) and the gold-rSjSAP4 conjugate (10 µg/ml) was applied onto conjugate pad (glass fibre membrane) at a volume of 35 μl/cm and dried in a biochemical incubator for 12 hours at 37 °C. The buffer used for conjugate pad preparation contains 20 mM Tris, 5% (w/v) sucrose, and 2.5% (w/v) trehalose. By using an XYZ Biostrip Dispenser (HM3030, Shanghai Kinbio Tech. Co., Ltd, Shanghai, China), 1 mg/ml Protein G (Zoonbio Biotechnology, Nanjing, China) and 0.7 mg/ml mouse anti-His tag mAb (Zoonbio Biotechnology, Nanjing, China) were dispensed onto the nitrocellulose (NC) membrane (CN140, Sartorius, Goettingen, Germany) (porosity: 8 μm, wicking rate: 110-165s/4cm) at a volume of 1 μl/cm to form the test and control lines, respectively. The membrane was then dried at room temperature in a biochemical incubator for 6 hours. The absorbent pad (filter paper) coated membrane (NC membrane), conjugate pad (glass fibre membrane), and sample pad (glass fibre membrane), were laminated and pasted onto a plastic-backed support card with a 1–2 mm overlap as illustrated in **Figure 1**. The entire assembled scale board was cut lengthwise and divided into strips measuring 3 × 60 mm using a guillotine cutter (ZQ2002, Shanghai Kinbio Tech. Co., Ltd, Shanghai, China). The resulting strips were assembled in a plastic cassette, which was further placed into a silica gel desiccant-containing aluminium foil bag, and stored at room temperature.

### 2.6 GICA assay measurement and analysis

For each test, 50 µl diluted serum sample was added to each cassette. The strips were scanned at 10 min after sample loading. The tests were determined as invalid when the control band did not appear or when the tests were left to develop for more than 15 min. All images were uploaded to a computer and analysed by a Java-based image processing program, ImageJ to quantify the intensity of the bands. In order to convert the results to fully quantitative information, an R value, which was defined as the intensity of the test band divided by that of corresponding control band, was introduced.

### 2.7 ELISA

The ELISA assay was performed as described previously (39). MaxiSorp high protein-binding capacity 96-well ELISA plates (Nunc, Roskilde, Denmark) were coated with 100 ng recombinant SjSAP4 in coating buffer (100 μl/well) overnight at 4°C. The plates were blocked with 1% (w/v) bovine serum albumin (BSA) in phosphate-buffered saline with 0.05% Tween-20 (PBST pH 7.4) for 1 h at 37°C. Serum samples diluted at 1:250 in blocking buffer were added to the wells and the plates were incubated for 1 h at 37°C. A mouse monoclonal anti-human IgG (Fc specific)-biotin antibody (Sigma-Aldrich Co, MO, USA) was then added as secondary antibody (1:20,000, 100 μL/well) for 1 h at 37°C. The plates were further incubated with Streptavidin-HRP (BD Pharmingen, CA, USA) (1:10,000, 100 μl/well) at 37°C for 0.5 h. Plates were washed 5 times with PBST after each step. Next, 100 μl 3,3′,5,5′-tetramethylbenzidine (TMB) substrate was added to each well to develop the colorimetric reaction, which was terminated after 5 min by adding 50 μl 2 M sulfuric acid per well. The ELISA plates were read at OD 450 nm with a microplate reader (BMG LABTECH, VIC, Australia).

### 2.8 Statistical analysis

To analyse the differences in R values of GICA tests with serum samples at different dilutions or with different dilution buffers, Student’s *t*-test was used. Cut-off values for the GICA assay and SjSAP4-ELISA assay were set with the maximization of Youden’s *J*-index. Pearson’s correlation coefficient (r) was used for the assessment of the correlation between the R values of the GICA assay and OD values of the SjSAP4-ELISA assay. Agreement between the GICA strip and the SjSAP4-ELISA assay was determined using the Kappa statistic (https://www.graphpad.com/quickcalcs/kappa1/). Statistical analyses were performed using GraphPad Prism version 9.4.0 software (GraphPad Software, Inc., San Diego, CA, USA). Statistics were considered significant at a *p*-value less than 0.05.

## 3 Results

### 3.1 The detectable limit of the GICA assay

**Figure 2:**
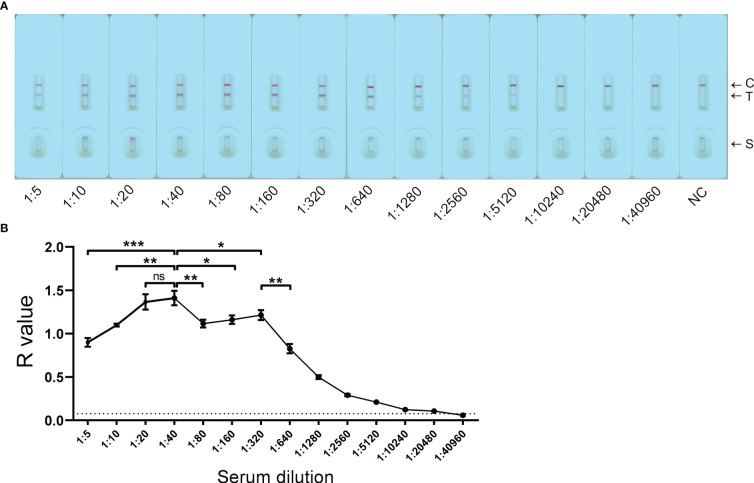
Detection limit of the GICA assay on pooled sera. **(A)** The GICA strips were tested with a pooled serum sample from KK-positive individuals (n = 3) with an EPG of 13, 14, and 20, respectively, at a series of dilutions (from 1:5 to 1:40960). C, Control line; T, Test line; S, Sample well; NC, a pooled serum sample from non-endemic controls (n = 3) tested at a dilution of 1:5. **(B)** R values of the GICA assay testing a pooled serum sample from KK (+) individuals (n = 3) at a series of dilutions. Dotted line: cut-off value determined as 2.1 times the mean R values of the pooled serum sample from non-endemic controls. The test was repeated in triplicate. *p* values were calculated using the Student’s *t*-test (ns, no significant difference; *p < 0.05; **p < 0.001; ***p < 0.001).

The developed GICA strips were tested with a pooled serum sample collected from KK-positive (KK (+)) individuals (n = 3) at a serial dilution (from 1:5 to 1:40960). The strips were scanned at 10 min after sample loading (**Figure 2A**). Based on R value analysis with a cut-off value of 0.076, the detectable limit of the GICA assay on testing the diluted serum samples was 1:20480. The established GICA strip showed the highest R value when the serum sample was tested at a dilution of 1:40. Six dilution ratios (from 1:10 to 1:320) had an R value higher than 1 (**Figure 2B**), indicating that a relatively wide range of dilution ratios may be applicable for the strip test. The hook effect was observed when serum samples diluted at 1:5 and 1:10 were tested (**Figure 2B**). A significant decrease in R value was observed when the sample was diluted to 1:640 and beyond (**Figure 2B**).

### 3.2 Optimal dilution buffer for the developed GICA assay

**Figure 3:**
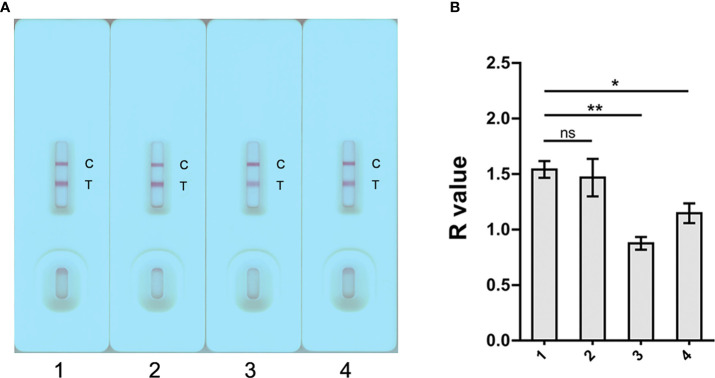
Determination of the optimal dilution buffer for the GICA assay. **(A)** Four dilution buffers, 1) PBS, 2) 0.9% NaCl, 3) 1% PBST, and 4) 2.5% sucrose containing 1% Tween 20, were selected to determine the optimal serum diluents. For each buffer, a pooled serum sample from three KK (+) individuals was tested in triplicate at a dilution of 1:40. **(B)** R value analysis revealed a significant impaired binding the gold-rSjSAP4 conjugate with antibodies in pooled serum samples when using PBST and 2.5% sucrose containing 1% Tween 20 as the dilution buffer compared with PBS. Data are represented as the mean ± SD from three different assays *p* values were calculated using the Student’s *t*-test (ns, no significance; *p < 0.05; **p < 0.01).

The *S. japonicum*-positive human serum samples were diluted with PBS, 0.9% NaCl, PBST (1% Tween 20) and 2.5% sucrose containing 1% Tween 20 in 1:40 to determine the best serum dilution buffer solution (**Figure 3A**). As shown in **Figure 3B**, PBS diluted sera showed a significant higher R value compared to sera diluted with PBST (1% Tween 20) (*p* < 0.01) and 2.5% sucrose (1% Tween 20) (*p* < 0.05); while there was no difference in R values when using PBS and 0.9% NaCl as the dilution buffer. The assay was performed in triplicate. PBS was then selected as serum diluent for the subsequent GICA tests.

### 3.3 Assessment of diagnostic performance of the GICA assay

**Figure 4:**
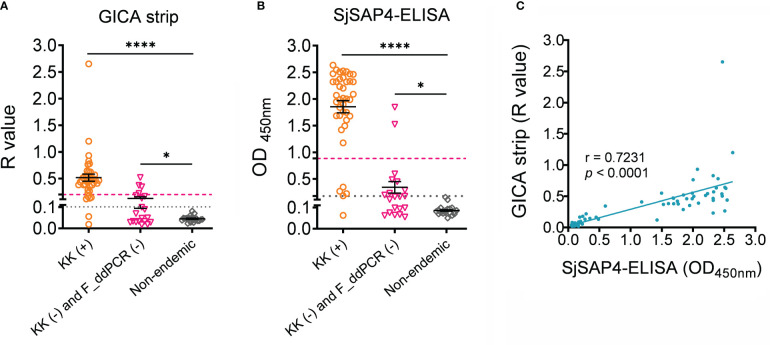
The performance of the SjSAP4-incorpated GICA and SjSAP4-ELISA assay in the diagnosis human schistosomiasis japonica. **(A, B)** Scatter plot showing the R values of the GICA assay (serum dilution 1:20) and OD values of the SjSPA4-ELISA assay (serum dilution 1:250) in testing serum samples from KK (+) individuals (n = 40), KK (-) and F_ddPCR (-) individuals (n = 20), and the non-endemic controls (n =20). Significance was analyzed by the Student’s *t*-test (****, *p* < 0.0001; *, *p* < 0.05). Gray dotted line: cut-off value determined using non-endemic group as control; Magenta dashed line: cut-off value determined using the KK (-) and F_ddPCR (-) group as control. **(C)** Correlation between the R values of the GICA strip and OD values determined by the SjSAP4-ELISA assay (n = 80) using Pearson’s correlation coefficient.

Serum samples collected from 80 subjects, including 40 KK (+) individuals, 20 endemic individuals confirmed as both KK-negative (KK (-)) and faecal droplet digital PCR assay-negative (F_ddPCR (-)) (18), and 20 healthy donors from a non-endemic area, were further tested with the GICA strips at a dilution of 1:20, given most of the KK (+) subjects harboring an EPG less than 10. Meanwhile, the SjSAP4-ELISA assay was performed on the same panel of serum samples. As shown in **Figures 4A, B**, individuals from the endemic areas had higher R values and OD values than non-endemic donors (*p* < 0.0001 and *p* < 0.05 for the KK (+) group, and KK (-) and F_ddPCR (-) group, respectively). Using the non-endemic group as control, the developed GICA strip showed a sensitivity of 95.0% and a specificity of 100% (**Figure 4A**), while the SjSAP4-ELISA assay had a sensitivity of 97.5% and absolute specificity (**Figure 4B**). Using the KK (-) and F_ddPCR (-) group as control, the GICA assay had a sensitivity of 85.0% and a specificity of 80.0% (**Figure 4A**), while the SjSAP4-ELISA assay displayed a sensitivity of 87.5% and a specificity of 90.0% (**Figure 4B**). A significant positive correlation between the developed GICA test and the SjSAP4-ELISA assay was observed (Pearson’s correlation, r = 0.7231, *p* < 0.0001) (**Figure 4C**). The Kappa statistics analysis indicated that there is an almost perfect agreement between the GICA strip and SjSAP4-ELISA assay (κ value = 0.964 and 0.893 using the non-endemic donors, and KK (-) and F_ddPCR (-) subjects as control, respectively).

### 3.4 Assessment of stability of the GICA strips

**Figure 5:**
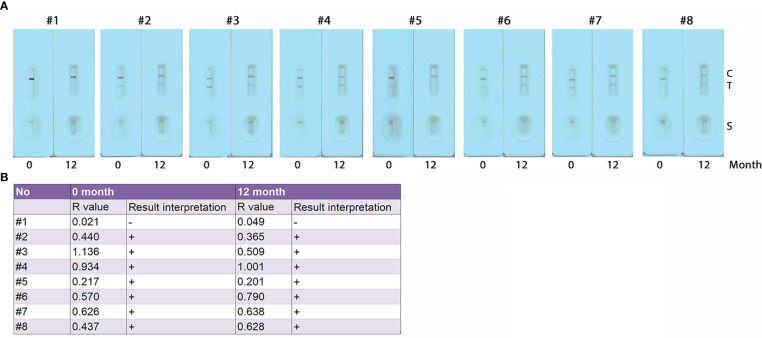
Stability assessment of the GICA strips. **(A)** Eight serum samples (one from healthy control (#1) and 7 from KK-positive subjects (#2 – #8)) diluted with PBS at 1:20 were used to assess the stability of the GICA strips stored at 25 for 12 months; **(B)** R values and read results for the eight serum samples tested with freshly produced GICA strips and those sealed and stored at 25°C for 12 months, respectively.

To establish the stability of the developed GICA strips, sealed cassettes were stored at room temperature and used at 12 months using serum samples collected from eight subjects, including one healthy control and seven KK (+) individuals (**Figure 5A**). R value analysis revealed that there was no difference in result interpretation between the freshly produced GICA strips and those sealed ones stored at room temperature for 12 months (**Figure 5B**). These results indicate that the GICA strips remain stable at least for 1 year when stored in the dark with desiccant at room temperature.

## 4 Discussion

In the Philippines, approximately 12 million Filipinos in 28 provinces across 12 geographical zones are potentially affected by schistosomiasis japonica, with two and half million directly exposed to the disease (43, 44). As of 2019, the national prevalence of the disease was reported to be 4.0% based on focal surveys (45). Nevertheless, the infection intensity of the disease is decreasing in endemic barangays of the Philippines where MDA has been implemented annually, and light infections with an EPG less than 99 are becoming more dominant (46). Development and deployment of rapid, easy-to-use, cost-effective, and field-deployable diagnostic tools represent an important component of an integrated and innovative control approach to achieve disease elimination. GICA is the most widely used POC diagnostic tool for the detection/screening of a variety of disorders/diseases, including infectious diseases (28, 47). In this study, we developed a novel recombinant protein-based GICA assay and assessed its performance in the diagnosis of human *S. japonicum* infection.

It has been noted that the application of SEA of *S. japonicum* or antigen purified from crude lysate of *S*. *mansoni* adult worms in GICA strip development caused significant cross-reaction with the antibodies to other parasitic flukes or soil-transmitted helminths, although showing a high sensitivity (28, 29, 48). Rodpai et al. recently developed an immunochromatographic test (Sj-ICT) for the diagnosis of schistosomiasis japonica using somatic extract from adult *S. japonicum* as target antigen (49). The diagnostic assay also showed cross-reactions with cases of *Opisthorchiasis viverrini*, *Clonorchiasis sinensis*, *Paragonimiasis heterotremus*, sparganosis, cysticercosis, trichinellosis and trichuriasis (49). These studies indicate that it is necessary to develop immunochromatographic tests by incorporating optimised recombinant antigens, such as tetraspanins, saposin family members and hepatic schistosomula antigens (30, 39, 50, 51), to minimise the potential risk of cross-reactions. In the current study, we used a *S. japonicum* saposin protein, SjSAP4 as the target antigen, which previously showed no cross-reaction with alveolar echinococcosis and trichinellosis in ELISA assays (41), for the development of GICA assay. In addition, a BLASTp search in the National Center Biotechnology Information (NCBI) database confirmed that there exist only homologous fragments sharing less than 30% sequence identity with SjSAP4 in other parasitic flukes, such as *C. sinensis* and *Fasciola hepatica*, making the potential cross-reactivity of SjSAP4 with these flukes less likely. Nevertheless, a further experimental validation of the developed SjSAP4-incorapted GICA strip with sera samples from patients infected with other helminths, particularly other parasitic flukes, is still needed to test the potential cross-reactivity.

Currently, there are mainly two types of design used for the development of GICA assays for the diagnosis of schistosomiasis. The first, as adopted by Xu et al. (10) and Shen et al. (29), the gold- or fluorescent protein-conjugated recombinant streptococcal protein G (rSPG) is added onto the conjugate pad, while the schistosome antigen and rSPG are immobilized on the NC membrane, as the T and C line, respectively. For the second design as adopted by Rodpai et al. (49) and Pearson et al. (30), the gold-conjugated mouse anti-human IgG was sprayed onto the conjugate pad, while the schistosome antigen and goat anti-mouse IgG were dispensed as the T and C line, respectively, on the NC membrane. The two designs require high concentration of schistosome-derived antigens to be sprayed on the NC membrane. However, the recombinant SjSAP4 exhibits limited solubility in non-denatured solution, restricting the feasibility to adopt the above designs in developing GICA assay incorporating SjSAP4. In addition, our previous study showed that there are limited linear B-cell epitopes available on SjSAP4, suggesting its antigenicity is predominantly dependent on conformational B-cell epitopes (52), a finding that excludes the use of denatured rSjSAP4 in the development of the GICA assay. In this study, a novel GICA format, as shown in **Figure 1**, was then adopted. Based on the design, it is possible that the non-specific antibodies may compete with the conjugated anti-SjSAP4 antibody complexes for binding to the immobilized protein G on the ‘T’ line, which may partly explain why serum samples at relatively high concentrations (such as those diluted at 1:5 and 1:10) did not show a greater R value compared to those at relatively low concentrations (such as those diluted at 1:20 and 1:40) when tested with the developed GICA strips (**Figure 2B**), in addition to the hook effect.

As the results of immunochromatographic strips need to be read within a limited time after sample loading, the result read must be conducted by well-trained investigators to achieve an accurate assessment if naked eye determination method is employed. There can, however, be variability in visual interpretation of trace results due to individual differences in visual acuity and/or training (53). In regarding of interpretation optimization, a reference colour card (34) or lateral flow reader (54) was employed in previous studies to increase accuracy in result reads. In this study, the results of the established GICA assay were timely scanned and further analyzed *in silico* to eliminate the inter-reader variability, a procedure similar to that undertaken previously (35, 55). By introducing an R value, we convert the results of the developed GICA assay into a fully quantitative test, which can minimise the likelihood of potential system errors caused by variations in the absorbance rate of samples and/or color development time among different cassettes.

The current GICA assay is an AbD based assay. As antibodies can persist in the host after parasite clearance, an inherent limitation of AbD assays is their limited ability to differentiate between past and current infection. In this regard, determination of antibody decay rates post-infection could be a relevant subject to determine diagnostic value for some applications. Previously, by employing a murine schistosomiasis model, we found that the levels of SjSAP4-specific IgG antibodies start to decline at 7 months post-chemotherapy in one out of six mice (39). However, murine schistosomiasis usually represents a high dosage of infection, which cannot fully reflect the authentic situation of the disease in humans. Thus, a similar investigation, i.e., exploring the decay of specific antibodies against SjSAP4 in schistosomiasis patients after chemotherapy would be expected in the future. Using non-endemic donors as controls, some individuals in the KK (-) and F_ddPCR (-) group were positive for the GICA assay (positivity rate: 35%) and SjSAP4-ELISA assay (positivity rate: 40.0%). The antibody reaction observed in these subjects indicates that they may have previously been infected with *S. japonicum* in the past (such as 3-12 months). It is worth noting that, the ability to discriminate between the current and previous infection can be improved for both assays by setting stringent cut-off values as we did in the case of individuals from endemic areas being used as controls, at the cost of sacrificing the diagnostic sensitivity to some extent. Nevertheless, the applications of such rapid and field-friendly POC tests for accurate diagnosis of schistosomiasis include: 1) Used as a screening tool for monitoring of transmission areas. For example, the developed GICA assay thus can be used for rapid mapping of schistosomiasis in the endemic zones in the Philippines, helping identify high-priority areas for targeted interventions. As the Department of Health (DOH) of the Philippines administers MDA for schistosomiasis every January through the Schistosomiasis Control and Elimination Program (56), the best time to carry out such rapid mapping should be at the end of the year. 2) Used as a screening tool for surveillance. In a limited prevalence or “post-elimination” situation, such as that in China, the GICA assay can also be used to determine if transmission blocking/elimination are actually achieved. 3) Testing individuals from non-endemic areas returning home after visiting schistosomiasis-endemic regions with suspicion of infection (travellers, migrants and international labourers), thereby helping prevent disease spread. 4) Determining the serological prevalence in endemic areas before and after the implementation of integrated interventions, thus monitoring the effect of these intervention measures. 5) Used to define the affected area of an *S. japonicum* infection focus, such as in Lindu, Napu, and Bada Highlands in Central Sulawesi, Indonesia (57), and two new endemic foci, Gonzaga and Calatrava, in the Philippines (58), by moving further away from the center until blood samples from surrounding areas are no longer positive.

As both are an indirect AbD based immunoassay, the SjSAP4-incorperated GICA and the SjSAP4-ELISA assay showed a high concordance. However, when the KK (-) and F_ddPCR (-) individuals were used as controls, the SjSAP4-incorperated GICA assay is inferior to the SjSAP4-ELISA assay in both sensitivity (85% vs 87.5%) and specificity (80% vs 90%), indicating that further optimisation steps are required to improve the performance of the immunochromatographic strip, such as validating the assay with serum samples at a higher dilution. Nevertheless, in contrast to GICA, the classic ELISA assay is labor-intensive and equipment-dependent, and requires a well-trained technician to perform. The stability assay of the GICA strips indicated that the validity period of the GICA strips was at least 12 months at room temperature, without loss of performance in the detection of *S. japonicum* infection. The GICA assay established here thus displays similar stability with the Sj-ICT and GICA assays previously developed by other groups (28, 49). In addition, due to high sensitivity of the established GICA strip, diluted finger-prick blood with a brief centrifugation to remove blood cells may also be suitable for the assay, which will further reduce the cost of the test.

## 5 Conclusions

In this study, a novel recombinant antigen-incorporated GICA assay was developed and assessed for the diagnosis of schistosomiasis japonica in subjects recruited from endemic areas in the Philippines. The application of the strip requires only a small serum volume and the results can be read within 10 min. The GICA strip showed a sensitivity of 95.0% and absolute specificity using the non-endemic individuals as controls. In addition, when the subjects confirmed as KK (-) and F_ddPCR (-) were employed as controls, the immunochromatographic test exhibited a sensitivity of 85.0% and a specificity of 80.0%. The GICA assay displayed a similar diagnostic ability with the conventional indirect ELISA method detecting serum IgG against rSjSAP4. The developed immunochromatographic strips are stable for at least for 12 months stored at room temperature in sealed aluminium foil bag with desiccant, without loss of performance. The GICA assay established here represents a powerful tool for large-scale screening in rural schistosomiasis japonica-endemic areas where access to facilities and supplies is limited.

## Data availability statement

The original contributions presented in the study are included in the article/**Supplementary Material**. Further inquiries can be directed to the corresponding author.

## Author contributions

YM, DM and PC formulated the research aims and prepared the study protocol. PC supervised the research. DM, AR, RO and PC, provided study materials. YM, DM, CG, HY, AR, RO and PC developed the methodology, collected and analyzed the data. YM and PC drafted the original manuscript. CG, AR and PC critically reviewed and edited the manuscript. All authors contributed to the article and approved the submitted version.

## Supplementary material

Supplementary Figure 1 Recombinant SjSAP4 protein expression and identification. **(A)** SDS-PAGE gel electrophoresis analysis of rSjSAP4 expression in *E. coli*. M, protein marker; lane 1, empty vector; lane 2-3, bacterial extract before and after rSjSAP4 expression, respectively; lane 4-5, supernatant and precipitate of bacterial extract expressing rSjSAP4, respectively. **(B)** SDS-PAGE analysis of purified rSjSAP4. M, protein marker; lane 1, BSA; lane 2, purified rSjSAP4. **(C)** Western blot analysis of rSjSAP4 using the Odyssey system. M, protein marker; lane 1, purified rSjSAP4 probed with an anti-His-tag antibody.

## References

[1] WHO. Ending the neglect to attain the sustainable development goals: a road map for neglected tropical diseases 2021–2030: overview. Geneva: World Health Organization (2020).

[2] McManus DP Dunne DW Sacko M Utzinger J Vennervald BJ Zhou XN. Schistosomiasis. Nat Rev Dis Primers (2018) 4(1):13. doi: 10.1038/s41572-018-0013-8 30093684

[3] Ross AG Olveda RM Chy D Olveda DU Li Y Harn DA. Can mass drug administration lead to the sustainable control of schistosomiasis? J Infect Dis (2015) 211(2):283–9. doi: 10.1093/infdis/jiu416 25070942

[4] Cavalcanti MG Silva LF Peralta RH Barreto MG Peralta JM. Schistosomiasis in areas of low endemicity: A new era in diagnosis. Trends Parasitol (2013) 29(2):75–82. doi: 10.1016/j.pt.2012.11.003 23290589

[5] Weerakoon KG Gobert GN Cai P McManus DP. Advances in the diagnosis of human schistosomiasis. Clin Microbiol Rev (2015) 28(4):939–67. doi: 10.1128/CMR.00137-14 PMC4548261 26224883

[6] Mesquita SG Caldeira RL Favre TC Massara CL Beck L Simoes TC. Assessment of the accuracy of 11 different diagnostic tests for the detection of schistosomiasis mansoni in individuals from a Brazilian area of low endemicity using latent class analysis. Front Microbiol (2022) 13:1048457. doi: 10.3389/fmicb.2022.1048457 36590409 PMC9797737

[7] Lv C Deng W Wang L Qin Z Zhou X Xu J. Molecular techniques as alternatives of diagnostic tools in China as schistosomiasis moving towards elimination. Pathogens (2022) 11(3):287. doi: 10.3390/pathogens11030287 35335611 PMC8951378

[8] Tabios IKB Sato MO Tantengco OAG Fornillos RJC Kirinoki M Sato M. Diagnostic performance of parasitological, immunological, molecular, and ultrasonographic tests in diagnosing intestinal schistosomiasis in fieldworkers from endemic municipalities in the Philippines. Front Immunol (2022) 13:899311. doi: 10.3389/fimmu.2022.899311 35774791 PMC9237846

[9] Coelho PM Jurberg AD Oliveira AA Katz N. Use of a saline gradient for the diagnosis of schistosomiasis. Mem Inst Oswaldo Cruz (2009) 104(5):720–3. doi: 10.1590/S0074-02762009000500010 19820832

[10] Xu B Gordon CA Hu W McManus DP Chen HG Gray DJ. A novel procedure for precise quantification of *Schistosoma japonicum* eggs in bovine feces. PloS Negl Trop Dis (2012) 6(11):e1885. doi: 10.1371/journal.pntd.0001885 23166847 PMC3499414

[11] Lindholz CG Favero V Verissimo CM Candido RRF de Souza RP Dos Santos RR. Study of diagnostic accuracy of helmintex, kato-Katz, and POC-CCA methods for diagnosing intestinal schistosomiasis in Candeal, a low intensity transmission area in northeastern Brazil. PloS Negl Trop Dis (2018) 12(3):e0006274. doi: 10.1371/journal.pntd.0006274 29518081 PMC5843168

[12] Gordon CA Acosta LP Gobert GN Olveda RM Ross AG Williams GM. Real-time PCR demonstrates high prevalence of *Schistosoma japonicum* in the Philippines: Implications for surveillance and control. PloS Negl Trop Dis (2015) 9(1):e0003483. doi: 10.1371/journal.pntd.0003483 25606851 PMC4301913

[13] Cnops L Soentjens P Clerinx J Van Esbroeck MA. *Schistosoma haematobium*-specific real-time PCR for diagnosis of urogenital schistosomiasis in serum samples of international travelers and migrants. PloS Negl Trop Dis (2013) 7(8):e2413. doi: 10.1371/journal.pntd.0002413 24009791 PMC3757062

[14] Mu Y Cai P Olveda RM Ross AG Olveda DU McManus DP. Parasite-derived circulating microRNAs as biomarkers for the detection of human *Schistosoma japonicum* infection. Parasitology (2020) 147(8):889–96. doi: 10.1017/S0031182019001690 PMC7391863 31840631

[15] Halili S Grant JR Pilotte N Gordon CA Williams SA. Development of a novel real-time polymerase chain reaction assay for the sensitive detection of *Schistosoma japonicum* in human stool. PloS Negl Trop Dis (2021) 15(10):e0009877. doi: 10.1371/journal.pntd.0009877 34695134 PMC8568117

[16] Weerakoon KG Gordon CA Gobert GN Cai P McManus DP. Optimisation of a droplet digital PCR assay for the diagnosis of *Schistosoma japonicum* infection: A duplex approach with DNA binding dye chemistry. J Microbiol Methods (2016) 125:19–27. doi: 10.1016/j.mimet.2016.03.012 27021661

[17] Weerakoon KG Gordon CA Cai P Gobert GN Duke M Williams GM. A novel duplex ddPCR assay for the diagnosis of schistosomiasis japonica: Proof of concept in an experimental mouse model. Parasitology (2017) 144(8):1005–15. doi: 10.1017/S003118201700021X 28274280

[18] Weerakoon KG Gordon CA Williams GM Cai P Gobert GN Olveda RM. Droplet digital PCR diagnosis of human schistosomiasis: Parasite cell-free DNA detection in diverse clinical samples. J Infect Dis (2017) 216(12):1611–22. doi: 10.1093/infdis/jix521 29029307

[19] Gandasegui J Fernandez-Soto P Carranza-Rodriguez C Perez-Arellano JL Vicente B Lopez-Aban J. The rapid-heat LAMPellet method: A potential diagnostic method for human urogenital schistosomiasis. PloS Negl Trop Dis (2015) 9(7):e0003963. doi: 10.1371/journal.pntd.0003963 26230990 PMC4521856

[20] Gandasegui J Fernandez-Soto P Muro A Simoes Barbosa C Lopes de Melo F Loyo R. A field survey using LAMP assay for detection of *Schistosoma mansoni* in a low-transmission area of schistosomiasis in umbuzeiro, Brazil: Assessment in human and snail samples. PloS Negl Trop Dis (2018) 12(3):e0006314. doi: 10.1371/journal.pntd.0006314 29534072 PMC5849311

[21] Garcia-Bernalt Diego J Fernandez-Soto P Febrer-Sendra B Crego-Vicente B Muro A. Loop-mediated isothermal amplification in schistosomiasis. J Clin Med (2021) 10(3):511. doi: 10.3390/jcm10030511 33535489 PMC7867102

[22] Guo Q Zhou K Chen C Yue Y Shang Z Zhou K. Development of a recombinase polymerase amplification assay for schistosomiasis japonica diagnosis in the experimental mice and domestic goats. Front Cell Infect Microbiol (2021) 11:791997. doi: 10.3389/fcimb.2021.791997 34869085 PMC8635165

[23] Rostron P Pennance T Bakar F Rollinson D Knopp S Allan F. Development of a recombinase polymerase amplification (RPA) fluorescence assay for the detection of *Schistosoma haematobium* . Parasit Vectors (2019) 12(1):514. doi: 10.1186/s13071-019-3755-6 31685024 PMC6827214

[24] Mesquita SG Lugli EB Matera G Fonseca CT Caldeira RL Webster B. Development of real-time and lateral flow recombinase polymerase amplification assays for rapid detection of *Schistosoma mansoni* . Front Microbiol (2022) 13:1043596. doi: 10.3389/fmicb.2022.1043596 36466644 PMC9716991

[25] MacGregor SR McManus DP Sivakumaran H French JD Gordon CA Cai P. Development of a novel CRISPR/Cas13-based assay for diagnosis of *Schistosoma japonicum* infection. medRxiv (2022). doi: 10.1101/2022.11.11.22282198

[26] Heidt B Siqueira WF Eersels K Dilien H van Grinsven B Fujiwara RT. Point of care diagnostics in resource-limited settings: A review of the present and future of PoC in its most needed environment. Biosensors (Basel) (2020) 10(10):133. doi: 10.3390/bios10100133 32987809 PMC7598644

[27] WHO. Public consultation: Target product profiles for diagnostic tests to meet schistosomiasis and soil-transmitted helminth programme needs. Available at: https://www.who.int/news-room/articles-detail/public-consultation-target-product-profiles-for-diagnostic-tests-to-meet-schistosomiasis-and-soil-transmitted-helminth-programme-needs (Accessed Jan 31, 2023).

[28] Xu R Feng J Hong Y Lv C Zhao D Lin J. A novel colloidal gold immunochromatography assay strip for the diagnosis of schistosomiasis japonica in domestic animals. Infect Dis Poverty (2017) 6(1):84. doi: 10.1186/s40249-017-0297-z 28388965 PMC5384140

[29] Shen Y Ji R Chai R Yuan N Zhang J Jing Y. A novel fluorescence immunochromatographic assay strip for the diagnosis of schistosomiasis japonica. Parasit Vectors (2021) 14(1):8. doi: 10.1186/s13071-020-04511-6 33407752 PMC7788720

[30] Pearson MS Tedla BA Mekonnen GG Proietti C Becker L Nakajima R. Immunomics-guided discovery of serum and urine antibodies for diagnosing urogenital schistosomiasis: A biomarker identification study. Lancet Microbe (2021) 2(11):e617–26. doi: 10.1016/S2666-5247(21)00150-6 PMC8683377 34977830

[31] Jiang SF Zhang XP Li BL He YY Liu J Tang YH. Evaluation of partially purified soluble egg antigens in colloidal gold immunochromatography assay card for rapid detection of anti-*Schistosoma japonicum* antibodies. Southeast Asian J Trop Med Public Health (2014) 45(3):568–75. 24974640

[32] Shen Y Wang Z Li J Xu R Ji R Lin J. Preparation of colloidal gold immunochromatographic test strips for the diagnosis of. Toxoplasma gondii. Food Agric Immunol (2020) 31(1):630–41. doi: 10.1080/09540105.2020.1749569

[33] Wang J He K Wu Z Jin W Wu W Guo Y. Development of a colloidal gold immunochromatographic strip for the rapid detection of antibodies against *Fasciola gigantica* in buffalo. Front Vet Sci (2022) 9:1004932. doi: 10.3389/fvets.2022.1004932 36187830 PMC9523912

[34] Sadaow L Rodpai R Janwan P Boonroumkaew P Sanpool O Thanchomnang T. An innovative test for the rapid detection of specific IgG antibodies in human whole-blood for the diagnosis of *Opisthorchis viverrini* infection. Trop Med Infect Dis (2022) 7(10):308. doi: 10.3390/tropicalmed7100308 36288049 PMC9607866

[35] Cai P Mu Y Weerakoon KG Olveda RM Ross AG McManus DP. Performance of the point-of-care circulating cathodic antigen test in the diagnosis of schistosomiasis japonica in a human cohort from Northern Samar, the Philippines. Infect Dis Poverty (2021) 10(1):121. doi: 10.1186/s40249-021-00905-5 34556183 PMC8460201

[36] Peralta JM Cavalcanti MG. Is POC-CCA a truly reliable test for schistosomiasis diagnosis in low endemic areas? the trace results controversy. PloS Negl Trop Dis (2018) 12(11):e0006813. doi: 10.1371/journal.pntd.0006813 30408030 PMC6224048

[37] Obeng BB Aryeetey YA de Dood CJ Amoah AS Larbi IA Deelder AM. Application of a circulating-cathodic-antigen (CCA) strip test and real-time PCR, in comparison with microscopy, for the detection of *Schistosoma haematobium* in urine samples from Ghana. Ann Trop Med Parasitol (2008) 102(7):625–33. doi: 10.1179/136485908X337490 18817603

[38] Graeff-Teixeira C Favero V Pascoal VF de Souza RP Rigo FV Agnese LHD. Low specificity of point-of-care circulating cathodic antigen (POCCCA) diagnostic test in a non-endemic area for schistosomiasis mansoni in Brazil. Acta Trop (2021) 217:105863. doi: 10.1016/j.actatropica.2021.105863 33587944

[39] Cai P Weerakoon KG Mu Y Olveda DU Piao X Liu S. A parallel comparison of antigen candidates for development of an optimized serological diagnosis of schistosomiasis japonica in the Philippines. EBioMedicine (2017) 24:237–46. doi: 10.1016/j.ebiom.2017.09.011 PMC5652020 28943229

[40] Cai P Weerakoon KG Mu Y Olveda RM Ross AG Olveda DU. Comparison of Kato Katz, antibody-based ELISA and droplet digital PCR diagnosis of schistosomiasis japonica: Lessons learnt from a setting of low infection intensity. PloS Negl Trop Dis (2019) 13(3):e0007228. doi: 10.1371/journal.pntd.0007228 30830925 PMC6417743

[41] Liu S Zhou X Piao X Hou N Shen Y Zou Y. Saposin-like proteins, a multigene family of *Schistosoma* species, are biomarkers for the immunodiagnosis of schistosomiasis japonica. J Infect Dis (2016) 214(8):1225–34. doi: 10.1093/infdis/jiw188 27190177

[42] Mu Y Weerakoon KG Olveda RM Ross AG McManus DP Cai P. Diagnostic performance of a urine-based ELISA assay for the screening of human schistosomiasis japonica: A comparative study. Front Microbiol (2022) 13:1051575. doi: 10.3389/fmicb.2022.1051575 36452928 PMC9703063

[43] Olveda RM Gray DJ. Schistosomiasis in the Philippines: Innovative control approach is needed if elimination is the goal. Trop Med Infect Dis (2019) 4(2):66. doi: 10.3390/tropicalmed4020066 31013917 PMC6631753

[44] Department of Health. Schistosomiasis control and elimination program (2018). Available at: https://doh.gov.ph/node/211 (Accessed Jan 13, 2023).

[45] Belizario VY Jr. de Cadiz AE Navarro RC Flores MJC Molina VB Dalisay SNM. The status of schistosomiasis japonica control in the Philippines: The need for an integrated approach to address a multidimensional problem. Int J One Health (2022) 8(1):8–19. doi: 10.14202/IJOH.2022.8-19

[46] Leonardo L Chigusa Y Kikuchi M Kato-Hayashi N Kawazu S-i Ma Angeles J. Schistosomiasis in the Philippines: Challenges and some successes in control. Southeast Asian J Trop Med (2016) 47(4):651–66.

[47] Li X Yin Y Pang L Xu S Lu F Xu D. Colloidal gold immunochromatographic assay (GICA) is an effective screening method for identifying detectable anti-SARS-CoV-2 neutralizing antibodies. Int J Infect Dis (2021) 108:483–6. doi: 10.1016/j.ijid.2021.05.080 PMC8180344 34091005

[48] Hoermann J Kuenzli E Schaefer C Paris DH Buhler S Odermatt P. Performance of a rapid immuno-chromatographic test (Schistosoma ICT IgG-IgM) for detecting *Schistosoma*-specific antibodies in sera of endemic and non-endemic populations. PloS Negl Trop Dis (2022) 16(5):e0010463. doi: 10.1371/journal.pntd.0010463 35622871 PMC9212132

[49] Rodpai R Sadaow L Boonroumkaew P Phupiewkham W Thanchomnang T Limpanont Y. Comparison of point-of-care test and enzyme-linked immunosorbent assay for detection of immunoglobulin G antibodies in the diagnosis of human schistosomiasis japonica. Int J Infects Dis (2021) 107:47–52. doi: 10.1016/j.ijid.2021.04.039 33864916

[50] Xu X Zhang Y Lin D Zhang J Xu J Liu YM. Serodiagnosis of *Schistosoma japonicum* infection: Genome-wide identification of a protein marker, and assessment of its diagnostic validity in a field study in China. Lancet Infect Dis (2014) 14(6):489–97. doi: 10.1016/S1473-3099(14)70067-2 24656567

[51] Hou N Piao X Jiang N Liu S Cai P Liu B. Novel hepatic schistosomula antigens as promising targets for immunodiagnosis and immunoprotection of schistosomiasis japonica. J Infect Dis (2022) 225(11):1991–2001. doi: 10.1093/infdis/jiac077 35235942

[52] Mu Y Gordon CA Olveda RM Ross AG Olveda DU Marsh JM. Identification of a linear B-cell epitope on the *Schistosoma japonicum* saposin protein, SjSAP4: Potential as a component of a multi-epitope diagnostic assay. PloS Negl Trop Dis (2022) 16(7):e0010619. doi: 10.1371/journal.pntd.0010619 35816547 PMC9302751

[53] Colley DG Andros TS Campbell CH. Schistosomiasis is more prevalent than previously thought: what does it mean for public health goals, policies, strategies, guidelines and intervention programs? Infect Dis Poverty (2017) 6(1):63. doi: 10.1186/s40249-017-0275-5 28327187 PMC5361841

[54] Mewamba EM Tiofack AAZ Kamdem CN Ngassam RIK Mbagnia MCT Nyangiri O. Field assessment in Cameroon of a reader of POC-CCA lateral flow strips for the quantification of *Schistosoma mansoni* circulating cathodic antigen in urine. PloS Negl Trop Dis (2021) 15(7):e0009569. doi: 10.1371/journal.pntd.0009569 34260610 PMC8312929

[55] Casacuberta M Kinunghi S Vennervald BJ Olsen A. Evaluation and optimization of the circulating cathodic antigen (POC-CCA) cassette test for detecting *Schistosoma mansoni* infection by using image analysis in school children in Mwanza Region, Tanzania. Parasite Epidemiol Control (2016) 1(2):105–15. doi: 10.1016/j.parepi.2016.04.002 PMC4946158 27430027

[56] DOH. Guidelines on the implementation of the harmonized schedule and combined mass drug administration (HSCMDA) for the prevention and control of lymphatic filariasis. schistosomiasis, and soil-transmitted helminths (DOH Memorandum No 2016-0212). (2016).

[57] Anastasia H Widjaja J. Engaging multi-sectoral collaboration to combat schistosomiasis in Napu Highlands, Poso District, Central Sulawesi. J Phys Conf Ser (2019) 1155(1):012111. doi: 10.1088/1742-6596/1155/1/012111

[58] Leonardo L Rivera P Saniel O Antonio Solon J Chigusa Y Villacorte E. New endemic foci of schistosomiasis infections in the Philippines. Acta Trop (2015) 141(Pt B):354–60. doi: 10.1016/j.actatropica.2013.03.015 23583862

