# Supplementary figures and images for: Development and assessment of a novel gold immunochromatographic assay for the diagnosis of schistosomiasis japonica
Source: Front Immunol. 2023 Apr 3;14:1165480. doi: 10.3389/fimmu.2023.1165480 (PMC10106775; doi:10.3389/fimmu.2023.1165480)

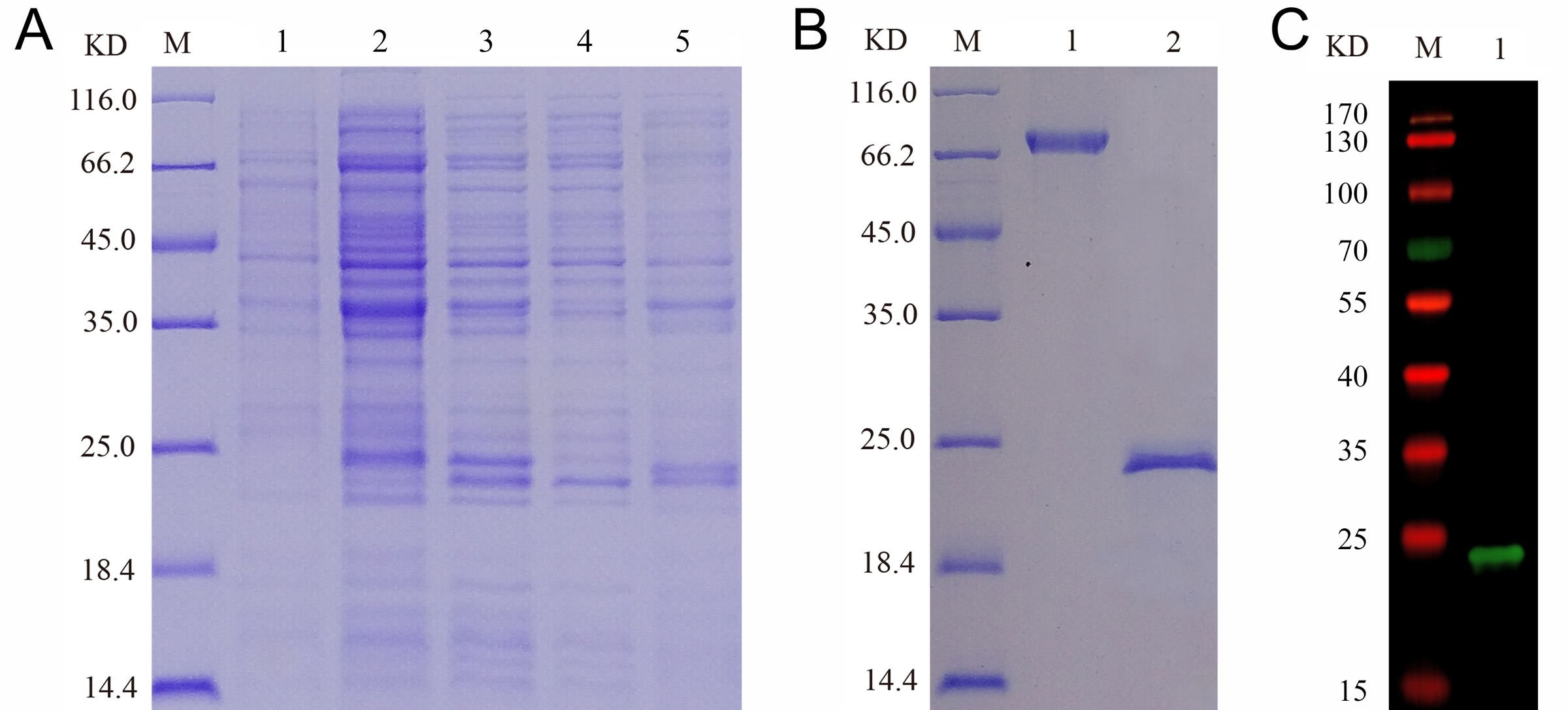

Supplement: Supplementary Figure 1 — Recombinant SjSAP4 protein expression and identification. (A) SDS-PAGE gel electrophoresis analysis of rSjSAP4 expression in E. coli. M, protein marker; lane 1, empty vector; lane 2-3, bacterial extract before and after rSjSAP4 expression, respectively; lane 4-5, supernatant and precipitate of bacterial extract expressing rSjSAP4, respectively. (B) SDS-PAGE analysis of purified rSjSAP4. M, protein marker; lane 1, BSA; lane 2, purified rSjSAP4. (C) Western blot analysis of rSjSAP4 using the Odyssey system. M, protein marker; lane 1, purified rSjSAP4 probed with an anti-His-tag antibody. [file Image_1.tif]
